# An epidemiologic survey on the causes of infertility in patients referred to infertility center in Fatemieh Hospital in Hamadan 

**Published:** 2015-08

**Authors:** Seyedeh Zahra Masoumi, Parisa Parsa, Nooshin Darvish, Sahar Mokhtari, Mahnaz Yavangi, Ghodratollah Roshanaei

**Affiliations:** 1 *Research Center for Child and Maternity Care (RCCMC), Midwifery Department, School of Nursing and Midwifery, Hamadan University of Medical Sciences, Iran.*; 2 *Chronic Disease Research Center, Mother and Child Health Department, School of Nursing and Midwifery, Hamadan University of Medical Sciences, Iran.*; 3 *Nahavand Social Security Clinic, Hamadan, Iran*; 4 *Malayer Mehr Hospital, Hamadan University of Medical Sciences, Iran.*; 5 *Endometrium and Endometriosis Research Center, Hamadan University of Medical Sciences, Iran.*; 6 *Modeling of Noncommunicable Diseases Research Center, Department of Biostatistics and Epidemiology, School of Public Health, Hamadan University of Medical Sciences, Hamadan, Iran.*

**Keywords:** *Male infertility*, *Female infertility*, *Etiology*

## Abstract

**Background:**

Infertility is considered as a major health care problem of different communities. The high prevalence of this issue doubled its importance. A significant proportion of infertility have been related to environmental conditions and also acquired risk factors. Different environmental conditions emphasized the need to study the different causes of infertility in each area.

**Objective:**

The aim of this study was to determine the frequency causes of infertility in infertile couples.

**Materials and Methods:**

In this cross sectional descriptive study 1200 infertile men and women that were referred to infertility clinic of Fatemieh Hospital during 2010 to 2011, were examined. This center is the only governmental center for infertility in Hamadan. Sampling was based on census method. Information about the patients was obtained from medical examinations and laboratory findings. To analyze the data, descriptive statistics such as frequencies and the mean were used.

**Results:**

The prevalence of primary and secondary infertility was 69.5% and 30.5% respectively. Among the various causes of infertility women factors (88.6%) had the highest regard. In the causes of female infertility, menstrual disorders, diseases (obesity, thyroid diseases, and diabetes), ovulation dysfunction, uterine factor, fallopian tubes and cervical factor had the highest prevalence respectively. The causes of male infertility based on their frequency included semen fluid abnormalities, genetic factors, vascular abnormalities, and anti-spermatogenesis factors, respectively.

**Conclusion:**

Etiology pattern of infertility in our study is similar with the many other patterns that have been reported by the World Health Organization. However, frequency of menstrual disorders is much higher than other studies that require further consideration.

## Introduction

Infertility means not having children after one year of regular sexual life without usage contraception techniques (1). Infertility is one of the major health care problems in all societies worldwide. The average prevalence of infertility in developed countries is 3.5-16.7% and in developing countries is 6.9-9.3% (2). The overall average of infertility was reported 13.2% in Iran (3). Causes of infertility are numerous such as anatomical, physiological and genetic factors. Many environmental and acquired factors also influence fertility and may lead to infertility. Menstrual and ovulation dysfunction and uterine factors are the most common causes of impairment in fertility. Etiology of infertility prevalence and patterns of causes of infertility in different regions are diverse. This discrepancy is due to existence of differences in environmental conditions associated with reproductive behaviors, such as age at marriage, environmental pollution, smoking and alcohol abuse, changing in lifestyle and diet (4). Although many studies have been conducted on the prevalence of infertility in the world, because infertility is increasing and the life style is changing and there is no comprehensive research in this area in Hamadan, it seems necessary to investigate the causes of infertility widely in Hamadan. Knowing the frequency of different causes of infertility in every region is important and can be effective in manager decisions. Due to the progress of methods of infertility treatment and the development of infertility treatment clinics in many cities of Iran, the majority of people within fertility problems after a while referred to these centers. Thus it seems that the infertile persons admitted to these centers can be a target population for the study of infertility in each region. In this study, different causes of infertility were examined in infertile couples.

## Materials and methods

The statistical population of this cross-sectional study was including 1200 infertile couples that referred to specialized infertility clinic of Fatemieh Hospital, Hamadan, Iran during 2010-2011. The vice chancellor for research and technology of Hamadan University of Medical Sciences approved the study (3841-35-16). Informed written consent was obtained from all individuals. In this study samples were selected by sampling census method. The infertile couples were examined and diagnostic tests were done on them. Inclusion criteria included the completeness and reliability of the results. For this purpose those who had completed the examinations were evaluated. And also the results were confirmed by specialists. Men and women were at reproductive age and had primary or secondary infertility. Exclusion criteria were lack of full examinations and laboratory tests.

Demographic characteristics of persons are recorded and each pair was visited by the gynecologist and specialist in kidney and urinary tract. For men, the information related to puberty, cryptorchidism inguinal herniorraphy, testicular viral inflammation, and sexually transmitted diseases have been documented through questioning. Physical examination of testes was done in order to varices network spermatic cord (varicocele) or other lesions. The index survey of sperm in the semen was performed according to World Health Organization guidelines. Hormonal investigation was done if necessary. About women, information about the changes of puberty, menstruation, abnormal milk exit from breast, sexual desire and sexual activity was being asked. Genital examination includes cervical stenosis, natural cervical mucus, and formation of endometriosis has been done. Hormonal evaluation was performed for all women. The ovulation was evaluated using ultrasonography and also the openness of tubes was examined using X ray of uterus and fallopian tubes. If necessary in some cases, laparoscopic device was used to examine peritoneal cavity. Secondary infertility refers to cases in which couples have experienced at least one pregnancy and then after a year of regular sexual life without contraception were unable to have children.

**Statistical analysis**

In order to analyze our data we employed the analytics software, SPSS (Statistical Package for the Social Sciences version 16.0, SPSS Inc., Chicago, IL, USA) and the Chi-square test was used. P<0.05 were considered to be statistically significant.

## Results

From 1200 infertile men and women, 834 cases (69.5%) suffered from primary infertility and 366 (30.5%) couples suffered from secondary infertility. Most infertile men (43.9%) were in the age group of 30-40 years and the majority of women (57.2%) were in the age group of 20-30 years. Average length of marriage of infertile couples was 91.6±63.8 month and their duration of infertility and sterility at the first visit in infertility clinic was 55.9±61.2 months. Overall 12.4% of couples had experience at least one of the assisted reproductive techniques such as micro injection, intra uterine infusion, in vitro fertilization, and zygote intrafallopian transfer. Of the total studied causes of infertility 88.9% was related to women factor and 66% was related to male factor. It should be noted that in some cases the couple may have more than one cause of infertility which overall all of the reasons have been investigated. Causes of infertility in women were as follows: menstrual disorders (disorders of cycle length and flow) 62.6%, diseases (obesity, thyroid diseases, diabetes) 58.7%, impaired ovulation (hormonal disorders, oligoovulation and anovulation) 50.3%, uterine causes 16.7%, tubal factor 15.4%, and cervical causes 7.9%. Abnormalities in uterine factors, hormonal dysfunctions in ovulation disorders, tubal obstruction in disorders of the fallopian tubes, cervical stenosis in cervical factor, dysfunction of cycle length in menstrual disorders, and obesity in diseases had the highest prevalence (Table I). In the male factor fertility there was semen abnormalities (44.6%), genetic factors (29.8%), anti-spermatogenesis agents (11%), and vascular disorders (17.2%). Varicocele in vascular abnormalities, asthenospermia in semen disorders, drugs in anti-spermatogenic agents, and oligoso-spermia less than 20 million per ml in genetic factors had the highest rates (Table II). After investigating the relationship between demographic characteristics with male and female etiological factors it was found that there was statistically significant relationship between the following variables: hormonal disorders with anemia (p=0.025), polycystic ovarian syndrome with increasing BMI (p <0.001), cervical stenosis and hirsutism and menstrual disorders with intake of medications prior to treatment of infertility (p <0.001), obesity with increasing duration of infertility (p=0.02), job and previous medications used in the treatment of infertility with varicocele (p <0.001), low sperm motility with increasing duration of infertility (p <0.001), low sperm count with residence (p <0.001), and duration of infertility with azoospermia (p<0.001). 

**Table I:** The frequency distribution of causes of female infertility in patients referred to Fatemieh Hospital in Hamadan

**Female causes** *	**Frequency**	**Percentage**
**Menstrual dysfunction**	751	62.6
**Diseases**	703	58.7
**Ovulatory dysfunction**	602	50.3
**Uterine**	199	16.7
**Tubal**	184	15.4
**Cervical**	95	7.9

Descriptive Test

* : Data are presented as n (%)

**Table II:** The frequency distribution of causes of male infertility in patients referred to Fatemieh Hospital in Hamadan

**Male causes** *	**Frequency**	**Percentage**
**Semen disorders**	536	44.6
**Genetic factors**	343	29.8
**Vascular disorders**	206	17.2
**Spermatogenesis agents**	83	11

Descriptive Test

* : Data are presented as n (%)

## Discussion

In this study, primary infertility was more common in infertile couples. These findings are similar to findings from other studies in this field in Iran (5-6) but are different from other studies that were conducted in the other parts of the world (7-8). The mean duration of infertility in this study was 5 years that was higher than the period of infertility in developing countries (9) and was comparable with some report that presented from some study (8, 10-11). In this study, the female factor had the highest percentage which is compatible with results of another study in Iran (12). Similarly in a study in Sari, Iran the most common cause of infertility was female factor (5). Conversely, in another study that was conducted in the Royan Institute, Tehran, Iran, the main cause of infertility was male factor (13). In most studies, male factor is reported 20-40%, female factor is 30-55%, combined factor is 35% and unknown etiology have been reported 5-15% (12, 14). The relatively large difference may be related to different conditions that may involve in infertility. In our study, uterine causes have been reported as a female factor for infertility. Although the uterine factor causes recurrent pregnancy loss and preterm delivery but in a study uterine anomalies in fertile women has been reported 2-3%, in infertile women 3% and in women with recurrent miscarriages it has been reported 5 to 10% (15). In our study, hormonal disorders had an important role in ovulation disorders. Hormonal disorders decreased ovarian reserve and even decrease the quality of the egg cell. In 5 to 10% of women lack of ovulation is due to low levels of gonadotropins and secondary estradiol is due to reduction in secretion of hypothalamic GNRH (16). In one study ovarian causes was the first infertility cause and tubal factor was the second factor in female infertility (5). In a study in Babol, Iran 26% of infertility causes was due to ovulation dysfunction (17). In our study, tubal obstruction in infertility has an important role in tubal factor infertility. Almost 20% of infertility in women is related to tubal disease that 10-25% of them are related to obstruction of the first part of tube (18). In a study in Sari, Iran, tubal factor was the second leading cause of infertility in women (19). Other cause of infertility due to the cervical factor was cervical stenosis in our study. Stenosis and closure of cervix are the rare causes of infertility (5). According to World health organization definition asthenospermia is decreased sperm mobility less than 50% of total sperm. Varicocele can lead to increase the temperature of testes and cause reflux of toxic metabolites of adrenal vein to the left kidney. Oligospermia means reduced number of sperms that are considered as infertility factor in male (15). 

In our study the major causes of infertility with male factors were varicocele, oligospermia and asthenospermia. In Karimpur and colleagues study in 2011, varicocele was responsible for 42.7% of cases of infertility in males (5). In a study in Shiraz, Iran, the most common cause of infertility in men attending infertility clinics was varicocele (20).

In reviews of infertile couples, study of male and female factors is important and mostly the couples mentioned more than one reasons as the infertility factor.
